# Natural Products from Marine Fungi—Still an Underrepresented Resource

**DOI:** 10.3390/md14010019

**Published:** 2016-01-16

**Authors:** Johannes F. Imhoff

**Affiliations:** GEOMAR Helmholtz Centre for Ocean Research Kiel, 24105 Kiel, Germany; jimhoff@geomar.de; Tel.: +49-431-600-4450; Fax: +49-431-600-4482

**Keywords:** marine fungi, marine natural products, *Tethya aurantium*, biological activities, fungal diversity

## Abstract

Marine fungi represent a huge potential for new natural products and an increased number of new metabolites have become known over the past years, while much of the hidden potential still needs to be uncovered. Representative examples of biodiversity studies of marine fungi and of natural products from a diverse selection of marine fungi from the author’s lab are highlighting important aspects of this research. If one considers the huge phylogenetic diversity of marine fungi and their almost ubiquitous distribution, and realizes that most of the published work on secondary metabolites of marine fungi has focused on just a few genera, strictly speaking *Penicillium*, *Aspergillus* and maybe also *Fusarium* and *Cladosporium*, the diversity of marine fungi is not adequately represented in investigations on their secondary metabolites and the less studied species deserve special attention. In addition to results on recently discovered new secondary metabolites of *Penicillium* species, the diversity of fungi in selected marine habitats is highlighted and examples of groups of secondary metabolites produced by representatives of a variety of different genera and their bioactivities are presented. Special focus is given to the production of groups of derivatives of metabolites by the fungi and to significant differences in biological activities due to small structural changes.

## 1. Introduction

For many years the study of marine fungi has been largely neglected for several reasons. One of the reasons may be related to the low abundance of fungi in the marine environment and a second with the doubt of the existence of true marine fungi. This has changed in recent years due to the recognition that marine fungi represent a quite diverse group and an excellent source of natural products. Despite the fact that many fungi are cosmopolitan and live as well in the sea as in other soil and freshwater habitats, in a number of cases we have obtained evidence that under the conditions of the marine environment, *i.e.*, in the presence of marine salts, a different metabolite profile is produced by fungi as compared to the fresh water situation [1,2,3]. This observation fits well with the general findings that changing the growth conditions is a good tool to promote the production of metabolites not seen under standard culture conditions. In addition to these cosmopolitan marine isolates, a group of true marine fungi exists that comprises a number of genera so far exclusively found in marine habitats. Cultivation-dependent studies demonstrated that marine macroorganisms, such as sponges and algae, are a rich source for fungi [2,4,5,6,7]. Even in deep-sea hydrothermal ecosystems, an unsuspected high diversity of fungal species was found using molecular approaches [8]. In consequence of this recognition, in recent years, an increasing number of new natural products have been characterized from marine fungi and there is no doubt that they produce a large number of interesting secondary metabolites, which often show pharmaceutically relevant bioactivities and may be candidates for the development of new drugs. By the end of the year 1992 only 15 fungal metabolites were reported [9] and approximately 270 compounds were described until 2002 [7]. In the period from 2000 to 2005, approximately 100 new marine fungal metabolites were listed [10] and this number increased to 690 in the period from 2006 until 2010 [6]. This trend still continues and members of the fungal genera *Penicillium* and *Aspergillus* were major objects in this field and they produced most of the described new compounds as depicted in recent reviews on this topic, e.g., by Blunt *et al.* [11]. However, the diversity of fungi in marine environments is by far not adequately represented in these studies on marine natural products. Though bioactivities of secondary metabolites from marine fungi reveal interesting levels for a number of clinical relevant targets, they are not well represented in the pipelines of drugs and none of them currently is on the market [12].

Therefore, we have put emphasis on the analysis of the fungal diversity of selected habitats and on the evaluation of the secondary metabolite production of a phylogenetically diverse selection of fungi from different marine environments. The overall goal was to reach out from the marine habitat to the candidate for drug development, as far as is possible in a research laboratory. The general strategy was to determine the cultured biodiversity and to translate this to the chemical diversity of natural products and to enforce biotechnological production of top candidates as outlined by Imhoff *et al.* [12]. An important aspect of this strategy is the use of a wide range of media and growth conditions to increase the cultured diversity of fungi, but also the variation of culture conditions for the isolates to improve the spectrum and the yield of produced metabolites. In addition, the following steps were of relevance for the success of this strategy: the identification of the isolates based on morphology and genetic sequence information as far as possible; the preservation of pure cultures in a strain collection, the extraction; dereplication, purification; identification and preservation in a physical substance library of secondary metabolites; the application of a wide range of bioactivity assays to the substances; and, if appropriate, the identification of chemical structures and improvement of their production by biotechnological methods [12].

The present report gives a personal view on work of marine fungi and their natural products of the authors group during the past 10 years and by no means attempts to give a comprehensive overview. Representative examples of biodiversity studies of marine fungi and of secondary metabolites produced by a diverse selection of marine fungi highlight major aspects of this research.

Our studies started up with interesting new natural products found in *Penicillium* species isolated from different marine sources and demonstrated the enormous potential of *Penicillium* and related common fungi. Later, a detailed analysis of the biodiversity of marine fungi from representative habitats was undertaken and the metabolite profiles of representative groups, including less abundant genera were analyzed. The large number of interesting new bioactive compounds found demonstrates the huge potential of marine fungi that have not been intensively studied so far.

In the following, we will: (i) firstly summarize results on recently discovered new secondary metabolites of *Penicillium* species; (ii) then highlight the diversity of fungi in selected marine habitats; and (iii) give examples of groups of secondary metabolites produced by fungi belonging to a wide range of genera, including their bioactivities.

## 2. Secondary Metabolites from *Penicillium* Species

Though representatives of *Penicillium* are among the most studied fungi and represent important drug producers, such as *Penicillium chrysogenum* (*P. chrysogenum*) as producer of penicillin and *Penicillium griseofulvum* as producer of griseofulvin, it is amazing how many new secondary metabolites continue to be found within this group of fungi as shown in reviews by Rateb and Ebel [6], Wang *et al.* [13] and Blunt *et al.* [11].

It happened that marine isolates of *P. chrysogenum* were our first intensively studied fungi, because they produced sorbicillacton A, which was considered as specifically active against human leukemia cell lines [14]. The work on *P. chrysogenum* revealed that in addition to sorbicillacton A and sorbicillacton B a number of other derivatives of sorbicillin were produced under the conditions applied. These included sorbicillin, 6-hydroxyoxosorbicillinol, oxosorbicillinol, sorbifuranol, sorbivineton and bisvertilonon [14]. Quite interestingly, the well-known product from this species, penicillin was not among the metabolites found under the applied conditions.

Studies on the biosynthesis and production of sorbicillacton A revealed that the compound was formed from sorbicillin, alanine and fumaric acid and always a small amount of sorbicillacton B (a saturated double bond in a side chain is the only structural difference) was formed. In order to achieve the production of larger amounts of sorbicillacton A, several approaches have been made. Because of serious problems in separating sorbicillacton A and B using HPLC methods, attempts were made to modify the culture conditions in such a way, that sorbicillacton A would be the only product. These attempts failed and always both sorbicillacton A and B were formed at a certain ratio. In order to increase the yield of sorbicillacton A, a selection of other marine isolates of *P. chrysogenum* was studied and, by using various culture media and growth conditions, strains were identified with strongly increased production rates of sorbicillacton A [1]. Quite interestingly, in addition to the culture conditions also the treatment of the spore suspensions used as an inoculum for the producing cultures turned out to be of importance for the production yield of sorbicillacton A. Most probably the history of the precultures and the treatment of spores have been underestimated in other similar studies. By choosing the most suitable spore suspension, the most appropriate culture conditions and the best producer strains, the yield of sorbicillacton A could be increased from initially 2–4 mg/L to more than 500 mg/L (and later even up to 1 g/L) [1]. However, the production was possible only in standing surface cultures with liquid media, but not in shaken cultures under submersed conditions. At the end, approximately 100 g of sorbicillacton A could be produced, purified and provided for further studies [1].

From the same fungus, the structure of sorbifuranone A was elucidated and a biosynthetic pathway proposed with a furanone as a precursor to become attached to sorbicillinol [15]. This cillifuranone was identified later in cell extracts of *P. chrysogenum*, which strongly supported the proposed biosynthetic pathway of sorbifuranone A [2].

In *Penicillium rugulosum*, more than 10 different derivatives of a polyene were produced and the chemical structures of some of these prugosines were determined [3]. These polyenes were typical polyketides formed from acetyl units and the methyl groups derived from the universal donor of methyl groups, *S*-adenosyl-methionine [3]. As they were rather unstable compounds, no detailed studies have been made regarding their biological activities.

Interesting compounds were also produced by the *Penicillium* species strain KF620 isolated from the North Sea and closely related to various *Penicillium* species (*P. verrucosum*, *P. viridicatum*, *P. hordei*, *P. tricolor*, *P. alii*, *P. albocoremium*, *P. neoechinulatum*) with 99% sequence similarity of 18S rRNA and ITS genes [16]. This strain produced several derivatives of eutypoids with good activity against glycogen synthase kinase 3β, a target for the treatment of diabetes 2. The eutypoids are unique structures which do resemble in three-dimensional appearance the synthetic compound 3B-415286 (though both compounds have significantly different structures), which is the result from a systematic search for a good inhibitor of GSK-3β [16,17]. Among the four tested eutypoid derivatives produced by the strain, in particular eutypoid B and C had good inhibitory activity (IC_50_: <1 µM) against glycogen synthase kinase 3β, if compared to the synthetic compound SB-415286 (IC_50_: 90 nM) [16].

These few examples demonstrate that marine isolates of the genus *Penicillium* still represent a good source for new and interesting bioactive compounds. It is quite remarkable to see that in all mentioned examples the producers built several derivatives of structurally related compounds at the time. Apparently, this is a general phenomenon also seen with other fungi as reviewed by Rateb and Ebel [6] and also shown in the examples below. This is especially important if one recognizes that the different derivatives in many cases have different bioactivity profiles and even may specifically act on a particular target system.

## 3. Fungal Diversity in the Marine Environment

Fungi are widely distributed in marine environments from the deep sea to polar ice covers. They occur in sediments and are found in all kinds of living and dead organic matter. Their numbers in ocean waters are quite low compared to bacteria and most of the studies on marine fungi have been made with those associated with marine sediments, with specific substrates like driftwood, algae, corals and in particular with sponges [2]. Quite a number of investigations have demonstrated that most marine sponges harbor a wealth of fungi, often with representatives of *Acremonium*, *Aspergillus*, *Fusarium*, *Penicillium*, *Phoma*, and *Trichoderma* [2,18]. Due to their accumulation within the animal a large number of fungal species can be isolated from sponges, which increases the probability to find representatives of less common taxa. For example, fungi belonging to the less common genera *Beauveria*, *Botryosphaeria*, *Epicoccum*, *Tritirachium*, and *Paraphaeosphaeria* have been obtained from marine sponges [5,19,20] and isolates belonging to *Bartalinia* and *Volutella* were obtained from *Tethya aurantium* [2].

In order to estimate the overall potential of marine fungi for natural product biosynthesis, it is important to know the phylogenetic diversity of marine fungi, the biosynthetic potential of the species and strains and the phylogeny of natural products biosynthesis. Therefore, much emphasis has to be given to the determination of the phylogenetic position of the fungi under investigation in order to enable correlation of the phylogenetic relationship to secondary metabolite production, respectively, to the genetic potential of such a production obtained from genomic sequences. Quite a number of genomic sequences of fungi are currently under way and those completed demonstrate an overall tremendous biosynthetic capacity of fungi, with commonly about 30 to 40 biosynthetic gene clusters coding for secondary metabolites in a single genome [21,22,23]. The majority of these gene clusters have not yet been correlated to their corresponding natural products, but the consequent analysis of these data and their correlation with produced metabolites and their biosynthetic pathways will certainly give a solid background for future phylogenetic considerations of biosynthetic pathways.

The problems related to taxonomy and species identification of marine fungi have a serious impact on the evaluation of fungal species diversity and in consequence also on the analysis of the evolution and diversity of biosynthetic pathways of their secondary metabolites. In fact, from a total of approximately 30 studies on secondary metabolites from fungi of the deep sea, one-third was identified as belonging to *Penicillium*, one-third to *Aspergillus* and the remainder was distributed to different other genera [13]. The high proportion of members of the genera *Penicillium* and *Aspergillus* may reflect the high abundance of representatives of these two genera in the samples. It causes problems to estimate the overall diversity of cultured fungi, because the probability to isolate fungi present only in low abundance is significantly reduced. Furthermore, it is disappointing to see, that only part of the fungi of these and other studies were identified on a species level. Only one-third of the studies gave a species name to the isolated fungus [13] and not in all cases this identity must be correct, because of the difficult assignment to species of fungi in general. For this reason, a number of fungi studied in respect to natural product biosynthesis remained unidentified at the species level as is depicted in several reports and reviews (e.g., [2,6,16]).

We have studied the diversity of fungi in the deep Mediterranean Sea and in marine sponges, in particular in *T. aurantium* [2] and evaluated the production of natural products from less abundant fungal genera. A valuable tool for the identification of fungal isolates proved to be the combination of morphological criteria and the comparison of the ITS1-5.8S-ITS2 fragment sequences, though even with these solid data, a clear species assignment was not always possible.

Our studies with sediments from the deep Mediterranean Sea indeed showed a dominance of *Aspergillus* and *Penicillium* isolates, which together represented almost half of all 43 isolates [24]. However, in addition, representatives from 10 other genera were found, including several isolates of *Cladosporium* and *Paecilomyces* and single isolates of *Acremonium*, *Auxarthron*, *Biscogniauxia*, *Capnobotryella*, *Engyodontium*, *Eutypella*, *Microascus* and *Ulcocladium*. The results of this study demonstrate that the majority of the cultured fungal genera are present in minor proportions and cannot be detected unless the number of isolates, unless the amount of sample used for isolation are significantly increased. Thus, a larger number of species and genera present in the studied sediments probably escaped detection due to the low number of (less than 50) isolates in total.

**Figure 1:**
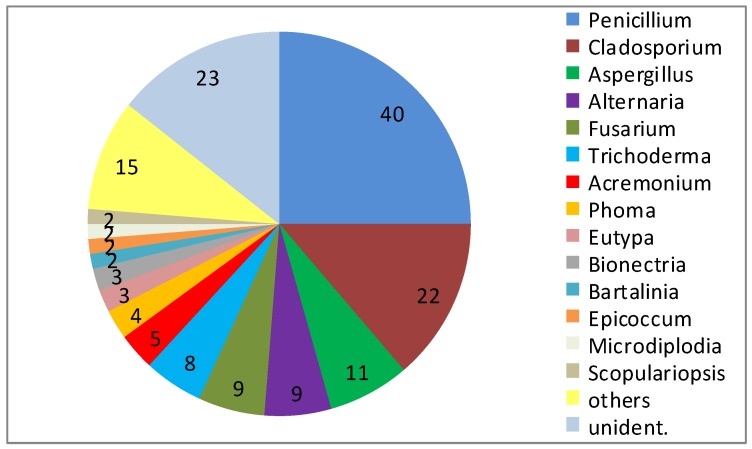
Diversity of fungal genera obtained from 10 specimen of *Tethya aurantium* from the Mediterranean Sea near Rovinj, with 29 identified genera among 160 isolates (numbers indicate the number of strains isolated).

Consistently, fungi isolated from sponges account for the largest number (28%) of novel compounds reported from marine fungi [7]. This high proportion may be related to both, the general interest in sponges as research objects and the abundance of fungi in sponges. In one of the most detailed studies on the cultured diversity of fungi associated with marine sponges, a particular high fungal diversity was obtained from *T. aurantium* and a detailed view on the isolated fungi revealed the presence of 29 genera among 160 isolates (23 not identified) (Figure 1) [2]. There was no clearly dominant group, either at the level of order or genus. Representatives of the following orders were isolated: *Eurotiales*, *Microascales*, *Hypocreales*, *Xylariales*, *Heliotiales*, *Capnodiales*, *Pleosporales* and *Botryosphaerales* [2]. Major groups included members of the genera *Penicillium* (40), *Cladosporium* (22), *Aspergillus* (11), *Alternaria* (9), *Fusarium* (9), and *Trichoderma* (8), but small numbers also of *Acremonium* (5), *Phoma* (4), *Eutypa* (3), and *Bionectria* (3) were present. In addition, 19 further genera (*Aureobasidium*, *Bartalinia*, *Engyodontium*, *Epicoccum*, *Eurotium*, *Gloetinia*, *Glomerella*, *Microdiplodia*, *Microdochium*, *Mucor*, *Myrothecium*, *Nectria*, *Paecilomyces*, *Petromyces*, *Peyronellea*, *Phoma*, *Pyrenochaeta*, *Verticillium*, and *Volutella*) were isolated, but were represented by one or two isolates only [2].

Because of the low abundance of many fungal genera, it is obviously necessary to increase the number of samples processed and of strains isolated to obtain a more complete view of their diversity. The high diversity seen at the genus level extends further to the subgenus-level. This is visible by the relatedness of strains in the phylogenetic trees based on sequences of 18S rRNA and ITS genes [2], in particular with those genera that are more abundant. The majority of isolates of *Aspergillus* and *Penicillium* as members of the *Eurotiales* and of *Alternaria* as member of the *Pleosporales* quite likely are not identical at the species level. In contrast, isolates of *Fusarium*, *Cladosporium* and *Trichoderma* include groups of closely related strains quite likely belonging to the same or closely related species [2]. Though the diversity based on 160 isolates is very high, a view on diversity and abundance of represented genera makes it very likely that proper attempts to further increase the number of isolates also will increase the depicted diversity at different taxonomic levels.

**Figure 2:**
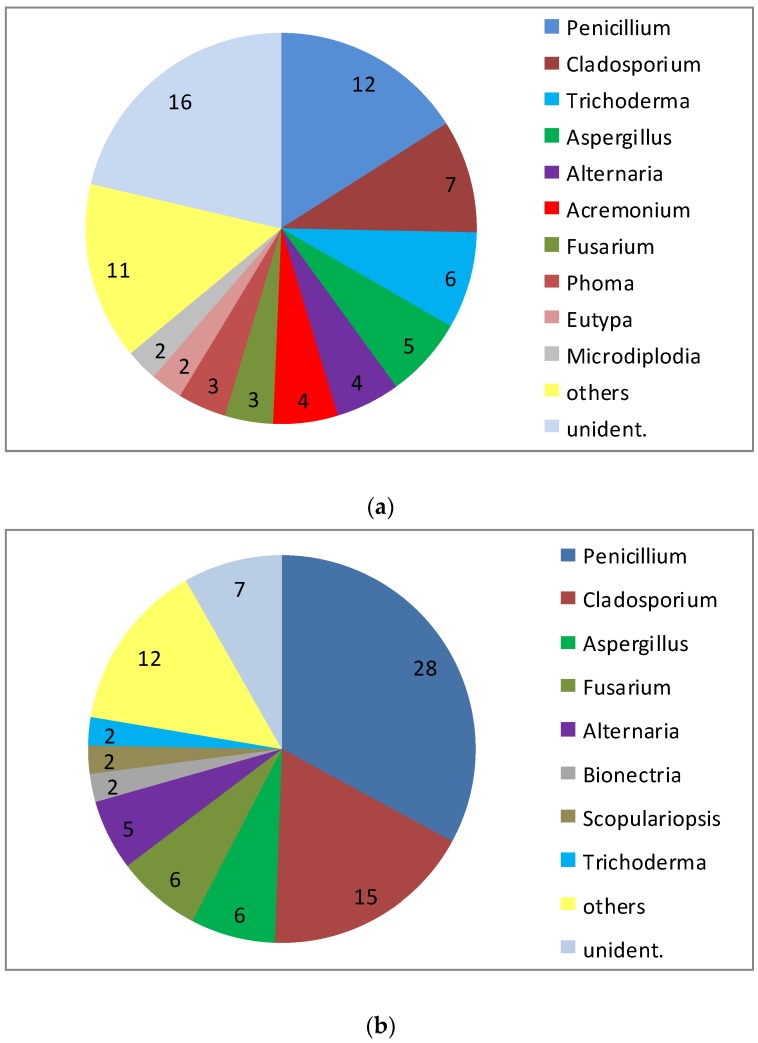
Diversity of fungal genera isolated from 10 specimen of *Tethya aurantium* from the Mediterranean Sea: (**a**) isolates from the cortex and (**b**) from the core part (numbers indicate the number of strains isolated).

A peculiarity of the sponge *T. aurantium* is the clear distinction of different types of cells in the outer cortex layer and in the inner core part of the sponge. Even more significant, the bacterial communities within the two parts of the sponge are different to the exclusion of common sequences [25]. This was reason to have a separate view on the fungal community of both the core part and the cortex of this sponge. An exclusive differentiation as seen for the bacteria could not be revealed (Figure 2). However, with a total of 85 isolates from the core part and 75 isolates from the cortex, it is obvious that the abundance of *Penicillium* in the cortex (approximately 16% of all) is much lower compared to the core part (approximately 33% of all). Though the total number of genera isolated from the two parts of the sponge were almost identical (21 *versus* 20), several genera were isolated only from one of the two compartments: *Aureobasidium, Gloeotinia, Microdiplodia, Nectria, Petromyces, Pyrenochaeta, Verticillium* and *Volutella* were only found in the cortex, while *Eurotium, Glomerella, Microdochium, Mucor, Myrothecium, Paecilomyces, Peyronella, Phoma* and *Scopulariopsis* were only obtained from the core part. However, because the low number of isolates from these genera (one or two) this result is considered to point out an incomplete recovery of the diversity in both compartments rather than to demonstrate clear differences in their composition.

In order to describe the chemical diversity of natural products formed by fungi obtained from *T. aurantium*, a selection of the isolates were screened for natural products and bioactive compounds, and many known metabolites were identified [2], no matter whether the strains were from abundant or rare genera. Among the described new structures were the scopularides from *Scopulariopsis brevicaulis* [26], the chlorazaphilones from *Bartalinia robillardoides* [27] and the cillifuranone from a *Penicillium* strain [2]. In addition, the most potent producer of sorbifuranones A–C was a *P. chrysogenum* isolated from *T. aurantium* [1,15].

## 4. New Metabolites from Marine Fungi

In order to obtain information on the metabolites produced from a wide range of marine fungi, we studied representative isolates from a number of different genera including less studied groups and genera isolated from various marine sources. Among the genera and species studied are *Asteromyces cruciatus* [23], *Trichoderma* sp. MF106 [28], *Stachybotrys* sp. MF347 [29], *Talaromyces* sp. LF458 [30], *S. brevicaulis* LF580 [26], *Calcarisporium* sp. KF525 [31,32], *B. robillardoides* LF550 [27], *Cladosporium* sp. KF501 [33], and *Massariosphaeria typhicola* KF970 [34]. A number of new compounds and their bioactivities from these studies were reported.

### 4.1. Trichoderma sp. Strain MF106

Fungi of the genus *Trichoderma* (order *Hypocreales*) are widespread in both terrestrial and marine environments. They are frequently found on decaying wood and in soil, as well as in marine sediments, marine sponges, and mangrove forests [35]. Marine representatives of the genus *Trichoderma* produce a variety of bioactive metabolites, such as the antimycobacterial aminolipopeptide trichoderins [36], the antifungal trichodermaketone A [35], the cytotoxic dipeptide trichodermamide B [37] and antibacterial tetrahydroanthraquinone and xanthone derivatives [38,39].

**Figure 3:**
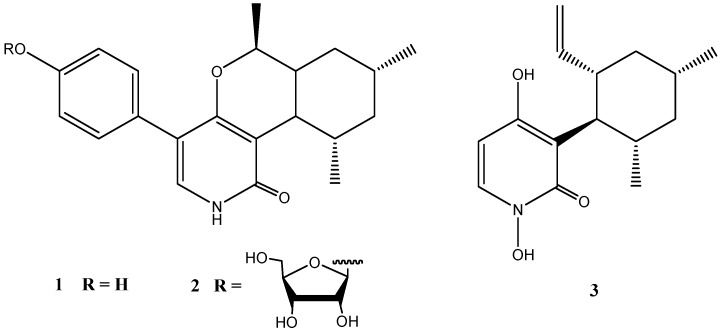
Chemical structures of compounds **1**–**3**.

Two unusual pyridones, trichodin A (**1**) and trichodin B (**2**), together with pyridoxatin (**3**) were produced by the marine *Trichoderma* sp. strain MF106 from the Greenland Sea [28]. Trichodin B (**2**) turned out to be a ribofuranoside of **1** and represented the first example of a pyridone with a glycosylated mono sesquiterpene (Figure 3).

Trichodin A showed moderate antibiotic activities against Gram-positive bacteria including the clinical relevant *Staphylococcus epidermidis* (IC_50_: 24 µM), but was not active against *Trichophyton rubrum.* Trichodin B exhibited no antimicrobial activity [28].

### 4.2. Stachybotrys sp. Strain MF347

The genus *Stachybotrys* (order *Hypocreales*) comprises approximately 100 species [40] and marine isolates have been obtained from various marine environments such as the rhizosphere of mangroves, mud of the intertidal zone, intertidal pools, brackish waters, marine sediments and sponges, marine algae, and sea fans [29,41].

**Figure 4:**
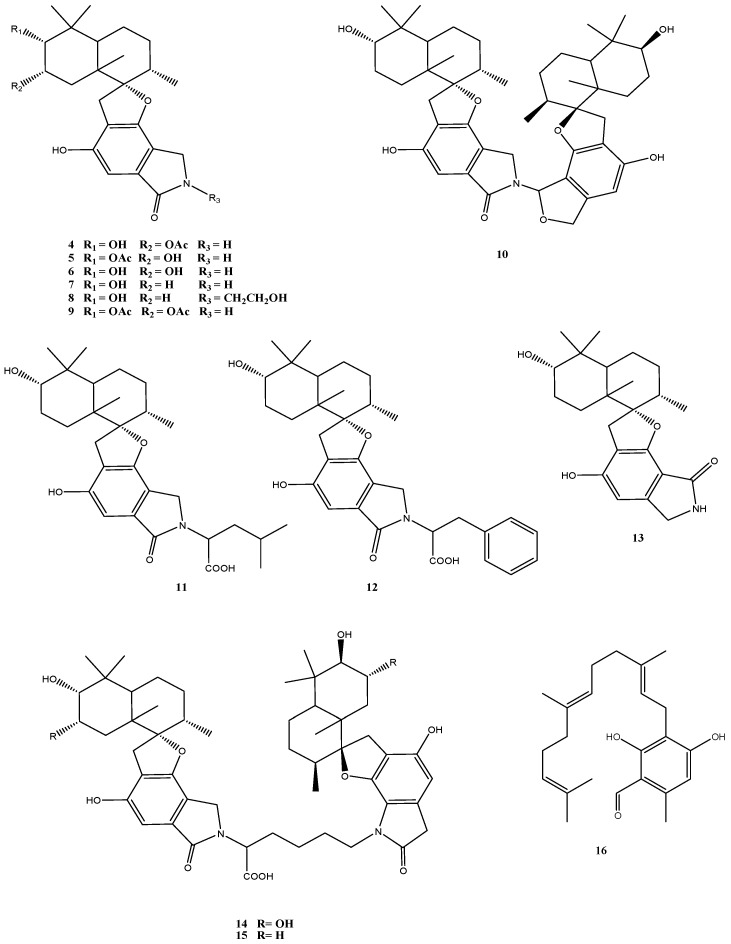
Chemical structures of compounds **4**–**16**.

A major class of secondary metabolites produced by *Stachybotrys* species, including isolates of *S. chartarum* obtained from habitats around the world is represented by spirocyclic drimanes [42]. Altogether 13 structurally and biosynthetically related compounds were identified in *Stachybotrys* sp. strain MF347 isolated from marine driftwood [29] (Figure 4). This fungus produced a number of known spirocyclic drimanes such as stachybocin A (**15**) and stachybocin B (**14**) featured by two sesquiterpene-spirobenzofuran structural units connected by a lysine residue, chartarlactam O (**13**), chartarlactam K (**5**), F1839A (**6**), stachybotrylactam (**7**), stachybotramide (**8**), and 2α-acetoxystachybotrylactam acetate (**9**), as well as the sesquiterpene ilicicolin B (**16**). The most conspicuous new metabolites of this strain were the spirocyclic drimanes stachyin A (**4**) and stachyin B (**10**) [29].

Quite a number of different biological activities are associated with spirocyclic drimanes, including immune-suppressive activity [43], endothelin receptor antagonistic activity [44], and inhibition of tyrosine kinase [45]. Clear differences were obtained in the bioactivities of spirocyclic drimanes with two and those with one sesquiterpene-spirobenzofuran structural units. While the spirocyclic drimanes of the first group (**10**, **14** and **15**) showed antibacterial activity against Gram-positive bacteria, including the clinically relevant methicillin-resistant *Staphylococcus aureus* (MRSA), those of the second group with one sesquiterpene-spirobenzofuran structural unit (compounds **4**–**9** and **11**–**13**) exhibited no activities [29]. Cytotoxic activity (IC_50_: 13–14 µM) was specifically found for stachyin B (**10**) only [29].

### 4.3. Talaromyces sp. Strain LF458

*Talaromyces funiculosum* (order *Eurotiales*) has a world-wide distribution and is common in all climatic zones with the possible exception of extreme cold habitats. It has frequently been isolated from various habitats, including estuarine sediments, salt marshes and a mangrove swamps [46]. A fungus isolated from the marine sponge *Axinella verrucosa* and tentatively classified as a *T. funiculosum* strain LF458 was investigated and found to produce several polycyclic compounds with common motifs.

*T. funiculosum* is known as producer of a number of bioactive compounds [30], such as the approved drug lovastatin, which inhibits the HMG-CoA (3-hydroxy-3-methylglutaryl-coenzyme A) reductase, an important enzyme in the biosynthesis of cholesterol [47]. In addition, secalonic acid D, a compound with cytotoxic activity, 11-desacetoxy-wortmannin, a fungicidal and anti-inflammatory metabolite, and helenin, being active against the swine influenza virus, were identified [48,49,50]. Further metabolites isolated from this fungus include mycophenolic acid, patulin, and 3-*O*-methylfunicone, which inhibit the growth of the fungus *Rhizoctonia solani* which is pathogenic to tobacco [51]. The closely related species *Talaromyces pinophilum* is producer of the antibacterial pinodiketopiperazine A and 6,7-dihydroxy-3-methoxy-3-methyl phthalide, which also exhibit lethal activity to brine shrimp *Artemia salina* [52]. Notable is that during co-cultivation of *T. pinophilum* with *Trichoderma harzianum* the production of the *Talaromyces* metabolites secopenicillide C, penicillide, MC-141, pestalasin A, and stromemycin were enhanced [53].

**Figure 5:**
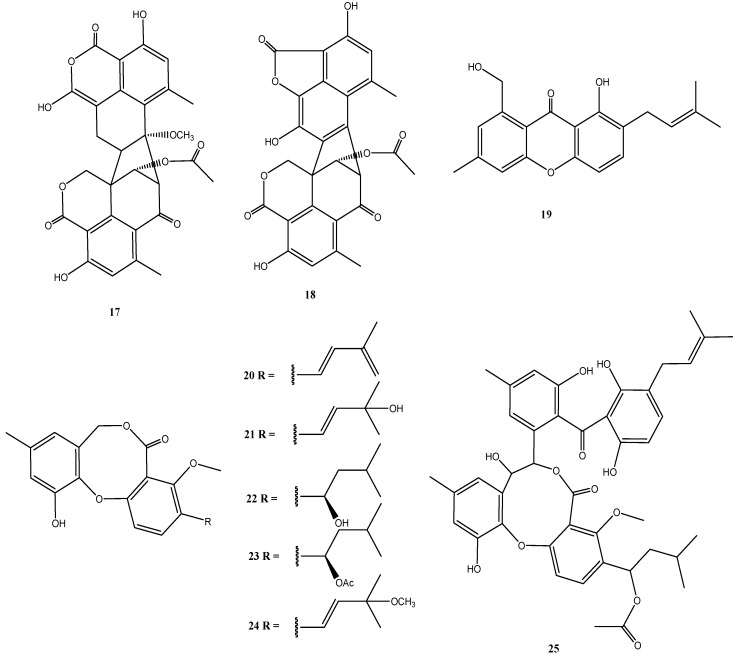
Chemical structures of compounds **17**–**25**.

In addition to two new oxaphenalenone dimers talaromycesone A (**17**) and talaromycesone B (**18**) and the new isopentenyl xanthenone talaroxanthenone (**19**), six diphenyl ether derivatives (Δ^1′,3′^,-1′-dehydroxypenicillide (**20**), 1′,2′-dehydropenicillide (**21**), vermixocin A (**22**), vermixocin B (**23**), 3′-methoxy-1′2′-dehydropenicillide (**24**) and AS-186c (**25**)) were identified in extracts from *Talaromyces* sp. strain LF458 (Figure 5).

Oxaphenalenones were isolated mainly from representatives of the genera *Talaromyces*, *Penicillium*, and *Coniothyrium* [54,55], including the antibacterial bacillosporins A–C from *Talaromyces bacillisporus* [54], the antibacterial and cytotoxic conioscleroderolide from *Coniothyrium cereal* [55], and erabulenols A and B from the *Penicillium* sp. FO-5637, which inhibit the cholesteryl ester transfer protein [56]. While oxaphenalenones have been previously obtained from *Talaromyces* species, talaromycesone B (**18**) represents the first 1-nor oxaphenalenone dimer carbon skeleton from a natural source. It should be noted, that, although Cao *et al.* [57] determined an *S*-configuration at C-9′ and made a generalizing conclusion that duclauxin and its analogues have this *S*-configuration, such a generalization is problematic and biosynthetically both epimers may be formed. This has to be taken into consideration with the structures of talaromycesone A and B (**17** and **18**).

The first oxaphenalenone with acetylcholinesterase inhibitory activity is represented by talaromycesone A (**17**). Also talaroxanthenone (**19**), and AS-186c (**25**) inhibited the activity of acetylcholinesterase (IC_50_: 1.6 µM to 2.6 µM) and these two compounds inhibited phosphodiesterase PDE-4B2 (IC_50_: 2.6–7.3 µM) in addition, but did not have antimicrobial or significant cytotoxic effects [30]. Compounds **17** and **25** also exhibited good antibacterial activities with IC_50_ values of 3.70 µM and 1.34 µM against human pathogenic *Staphylococcus* strains.

As new or more effective molecules for the treatment of Alzheimer’s disease are urgently needed, because the cases of this disease are expected to dramatically increase over the coming decades, compounds such as **17**, **19** and **25** are important as possible candidate molecules for drug development to treat neurological disorders. Their special advantage is the lack of cytotoxic properties. In addition, some of these molecules may have antibiotic properties (compounds **17** and **25**) against pathogenic bacteria [30].

### 4.4. Calcarisporium sp. Strain KF525

Fungal species of the genus *Calcarisporium* (order *Hypocreales*) have a widespread occurrence and are frequently found as mycoparasites or symbionts of higher basidomycetes and ascomycetes [58,59,60,61,62]. Among the natural products that have been described from this genus are antifungal compounds like 15-azahomosterols, aurovertins inhibiting the mitochondrial ATPases, and calcarisporins B_1_–B_4_ with calcarisporin B_1_ showing cytotoxic activity as discussed by Silber *et al*. [31].

**Figure 6:**
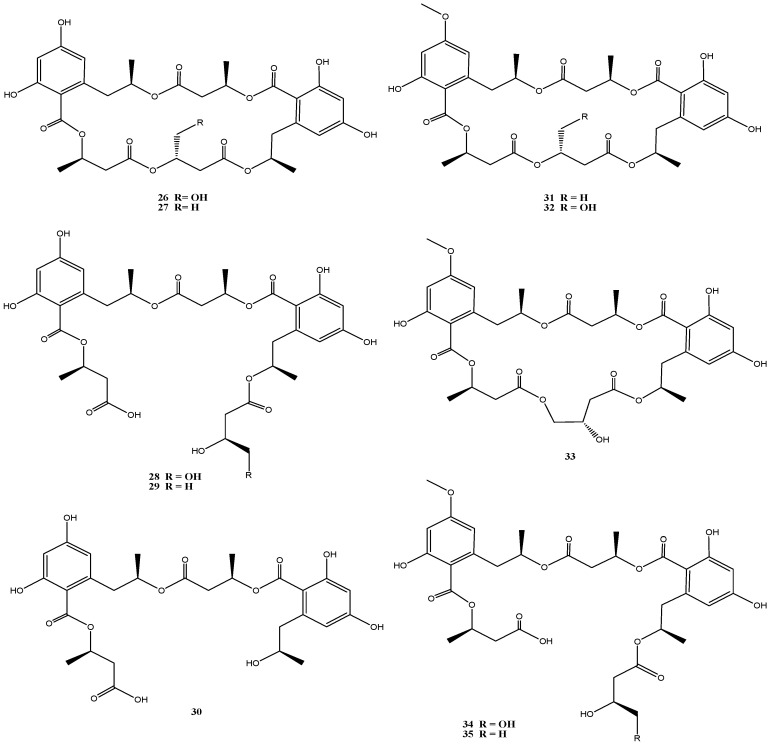
Chemical structures of compounds **26**–**35**.

An isolate from the German Wadden Sea, *Calcarisporium* sp. strain KF525, showed a diverse chemical profile, including three calcaripeptides, cyclodepsipeptides with highly common structural motifs [31] and ten structurally closely related linear and macrocyclic polyesters of the 15G256 group (15G256α, α-2, β, β-2 and π, **26**–**30**) and related (methylated) calcarides A–E (**31**–**35**) (Figure 6) [32]. The 15G256-type compounds are known as metabolites from a number of fungi, of ascomycetes (*Hypoxylon*, *Penicillium*, *Talaromyces*, *Acremonium* and *Scedosporium* species) as well as of the basidiomycete *Albatrellus confluens* [63,64,65,66,67,68].

Biological activities assigned to the 15G256 agents include antifungal, estrogenic and cytotoxic properties. They were also shown to potentiate nerve growth factor-induced neurite outgrowth. Compounds 15G256α (**26**) and β (**27**) for example attracted attention in the field of crop protection, as they displayed antifungal properties against the important plant pathogenic fungi, *Botrytis cinerea* and *Monilinia fructigena* [64].

The 15G256 compounds and the methylated derivatives thereof were found to display antibacterial activities with a clear structure-activity relationship [32]. Slight structural variation by methylation had significant influence on this activity. All macrocyclic compounds (**26**, **27**, **31**–**33**) inhibited *S. epidermidis* and *Xanthomonas campestris*, while the linear polyesters did not [32], indicating that the ring structure is required for this activity. Similar observations have been made for antifungal properties of the 15G256 compounds [63]. Although low to moderate antibiotic activity against *S. epidermidis* and *X. campestris* was associated with several of these compounds, a strongly reduced activity against *S. epidermidis* (but not against *X. campestris*) was found with the methylated analogs, and the strongest inhibition against *S. epidermidis* was exhibited by 15G256α (**26**) (MIC 13 µM). Good inhibitory activity against the *Xanthomonas* was restricted to the methylated calcaride A (**31**) (IC_50_: 6 µM) and inhibition of *Propionibacterium acne* was exhibited only by the non-methylated 15G256π (**30**) (IC_50_: 14 µM) [32].

### 4.5. Bartalinia robillardoides Strain LF550

The genus *Bartalinia* (order *Xylariales*) is rare among marine fungi and also is a rare genus among the isolates from the Mediterranean sponge *T. aurantium* [2]. *B. robillardoides* is known as a producer of taxol, an anticancer drug in clinical application [69].

**Figure 7:**
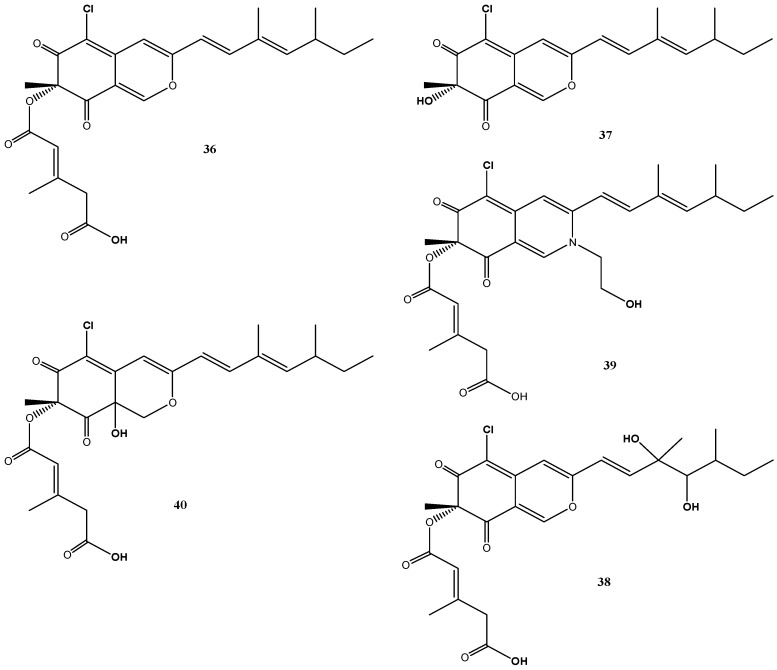
Chemical structures of compounds **36**–**40**.

*B. robillardoides* strain LF550 produced a number of secondary metabolites belonging to the chloroazaphilones. In addition to the known helicusin A (**36**) [70] and deacetylsclerotiorin (**37**) [71], three new chloroazaphilones, helicusin E (**38**), isochromophilone X (**39**) and isochromophilone XI (**40**), were identified (Figure 7) [27].

Though azaphilones represent a widespread family of fungal pigments and more than 170 azaphilones are produced by 23 different genera from 13 fungal families [72], they were for the first time described from the genus *Bartalinia* by Jansen *et al.* [27]. In particular the chlorinated congeners are less common and these have been found so far in representatives of *Penicillium*, *Chaetomium*, *Emericella*, *Talaromyces* and *Fusarium* [72].

Despite their structural similarities, the chlorazaphilones produced by this fungus revealed different biological activity spectra against a test panel of four bacteria, three fungi, two tumor cell lines and two enzymes [27]. Weak antibacterial activities were found against *Bacillus subtilis* and *Staphylococcus lentus* for compounds **37** and **40**. Antifungal activities (*Candida albicans*, *Trichophyton rubrum* and *Septoria tritici*) were found for deacetylsclerotiorin (**37**) (IC_50_: 2–10 µM) and for helicusin A (**36**), but not for helicusin E (**38**) and the methylester of helicusin A [27]. Most significant inhibition of acetylcholinesterase was associated with helicusin A (**36**) (IC_50_: 2.1 µM) and of phosphodiesterase PDE4 with deacetylsclerotiorin (**37**) (IC_50_: 2.8 µM) [27]. Interestingly, none of these compounds showed cytotoxic activity.

### 4.6. Cladosporium sp. Strain KF501

The genus *Cladosporium* (order *Capnodiales*) represents one of the largest and most heterogeneous fungal genera [73] with ubiquitous occurrence, including marine habitats. *Cladosporium* sp. strain KF501 was isolated from the German Wadden Sea and according to ITS sequence similarities was identical (100% similarity) to *Cladosporium cladosporioides*, *C*. *pseudocladosporioides*, *C*. *uredinicola*, *C*. *bruhnei* and *C*. *colombiae*. Hence, strain KF501 could be clearly identified as a member of the genus *Cladosporium*, but identification to the species level could not be achieved [33].

*Cladosporium* species have been shown to produce a variety of natural products, such as the melanins which are giving the fungal colonies their typical colored appearance, the antifungal cladosporides [74,75], the plant growth factors cotylenins [76,77], calphostins which specifically inhibit the protein kinase C [78], and cladosporin exhibiting a broad activity spectrum including antifungal, antibacterial, insecticidal, phytotoxic and immunosuppressive properties [79,80,81,82,83].

**Figure 8:**
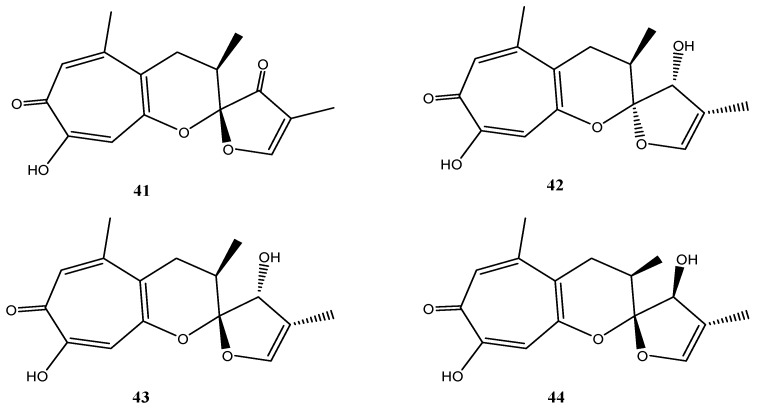
Chemical structures of compounds **41**–**44**.

Malettinins are tropolone/dihydropyran ring structures and are unique with regard to their linkage to a furan ring. Originally, malettinins A–C and malettinin D were reported from an unidentified fungus [84,85] and for the first time *Cladosporium* sp. strain KF501 was shown to produce malettinins A–C (**41**–**43**) and the new malettinin E (**44**), which is the 13-epimer of malettinin C (**43**) (Figure 8) [33]. Malettinin D was not found [33].

Malettinins A–C (**41**–**43**) displayed weak inhibitory properties against *C*. *albicans*, *B*. *subtilis* and *S*. *aureus* and malettinin A inhibited *Aspergillus flavus* and *Fusarium verticillioides* in addition [84]. Higher activities were found against the bacterium *X. campestris* and the fungus *T. rubrum* [33]. A clear influence of the chemical structure and of configurational changes on biological activities was observed. All stereoisomeric compounds (**42**–**44**) inhibited *X*. *campestris* in comparable concentration ranges, while **41**, possessing a very slightly modified furan ring, did not show inhibition. Consequently, it was suggested that the furan ring structure is critical for antibacterial properties of the malettinins against *X*. *campestris* [33]. In addition, activities against *T*. *rubrum* apparently are sensitive to configurational changes of the malettinins because the stereoisomers of the malettinins exhibited different IC_50_ values [33].

### 4.7. Massariosphaeria typhicola Strain KF970

Lindgomycetaceae (order *Pleosporales*) represent a less studied fungal group. Members of the Lindgomycetaceae were isolated from submerged parts of decaying wood and plant material in freshwater environments. Little is known on the metabolic capabilities of Lindgomycetes and related fungi and on their production of bioactive compounds.

Two fungi of the family Lindgomycetaceae, strains KF970 and LF327, were isolated from marine habitats of the Baltic Sea and the Arctic Ocean and were identified on the basis of 18S rRNA gene sequences. These were highly similar to each other and to *M. typhicola* (99.9% for KF970 and 99.5% for LF327) [34]. Therefore, both isolates can be regarded as strains of this species.

**Figure 9:**
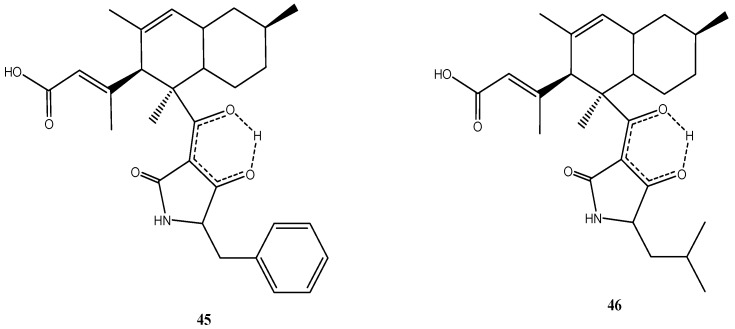
Chemical structures of compounds **45**–**46**.

Although both strains originated from different geographic regions, they produced the same metabolites. Two unusual antibiotic polyketides, lindgomycin (**45**) [34] and ascosetin (**46**) [86], were identified (Figure 9). They contain two distinct structural domains, a bicyclic hydrocarbon and a tetramic acid, which are connected by a bridging carbonyl. Naturally occurring tetramic acid derivatives originating from a variety of marine and terrestrial fungi have attracted a great deal of interest due to their broad-spectrum biological activities and challenging structural complexity [87,88]. The majority of the compounds isolated to date exhibited antibiotic or antiviral activity. Tetramic acids possessing an octahydronaphthalene skeleton also are active against Gram-positive bacteria but are rare in nature [87].

Lindgomycin (**45**) and ascosetin (**46**) revealed good antibiotic activity against a number of Gram-positive bacteria (IC_50_: 2–6 µM), the yeast *C. albicans* and the fungus *S. tritici* (IC_50_: 5–10 µM) [34]. Both compounds showed antibiotic activities against methicillin resistant *S. aureus* with IC_50_ values of 5.1 µM and 3.2 µM, respectively. The causative agents of black rot in crucifers (e.g., cabbage) and of leaf spot disease on crops (e.g., wheat), *X. campestris* and *S. tritici*, were also inhibited by the two compounds. No inhibition of Gram-negative bacteria was observed [34]. The lack of activity against Gram-negative bacteria already has been noticed for other tetramic acid derivatives [89] and maybe related to the difference in the cell wall structures. Apparently the outer membrane of Gram-negative bacteria is an efficient permeability barrier for these molecules.

## 5. Conclusions

The marine sponge *T. aurantium*, taken as a representative example of a suitable habitat for marine fungi, gives rise to assume that the fungal diversity in marine habitats is much larger than anticipated from those fungi studied so far in regard to the production of natural products. Due to the normally low number of fungi in marine habitats (compared to bacteria), however, it requires special effort to isolate a larger number of fungi from a particular sample. This in turn is necessary to adequately depict the natural diversity among the cultured strains. Nonetheless, the frequency of finding new secondary metabolites from marine fungi remains high. In addition, the following few key statements highlight the attractiveness of marine fungi as research object to search for new natural products.

- Despite the fact that natural products from some fungal genera, in particular *Penicillium* and *Aspergillus*, have been often and intensively studied, there is still a great potential of secondary metabolites produced by these fungi, which has not yet been fully explored.
- Much of the fungal diversity and the large potential of secondary metabolites of the untapped diversity of fungi in the marine environment still is to be discovered.
- Much of the genomic/genetic potential of secondary metabolites of cultured fungi is not produced under standard culture conditions. In particular, changes in media and culture conditions often change the metabolite profiles and are likely to increase the number of known products from the fungi. In addition, other methods such as cocultivation with other fungi or bacteria or the use of epigenetic modulators may stimulate biosynthesis of the “hidden genomic potential”.
- It has been demonstrated that the production of “families“ of secondary metabolites of structurally related derivatives, which often reveal significant differences in bioactivities, is common to many fungi. Because already small structural changes can be highly effective in regard to the bioactivity, it is important to study the full spectrum of structural variation offered by the fungi in order to unravel their biotechnological potential.

For improved success in future studies on secondary metabolites of marine fungi, the following points appear of primary importance:

- The multitude of natural derivatives of individual fungi and their bioactivities should be carefully explored.
- Biosynthetic pathways and their regulation need to be studied to conclude on explanations for the formation of multiple derivatives.
- The evolution of biosynthetic pathways of secondary metabolites should be systematically explored in respect to the presence of new biosynthetic gene clusters, in particular by use of the growing resource of fungal genome sequences.
- Genus- and species-specific metabolite profiles need to be elaborated using metabolomic and genomic approaches.

If these approaches and strategies are systematically applied to the search for secondary metabolites from cultures of marine fungi, we expect to receive a full treasure box of new bioactive natural products, some of which may qualify as excellent candidates for drug development.
